# Plasmonics with Metallic Nanowires

**DOI:** 10.3390/ma12091418

**Published:** 2019-05-01

**Authors:** Joanna Niedziółka-Jönsson, Sebastian Mackowski

**Affiliations:** 1 Institute of Physical Chemistry, Polish Academy of Sciences, 01-224 Warsaw, Poland; 2 Baltic Institute of Technology, 81-451 Gdynia, Poland; 3 Institute of Physics, Faculty of Physics, Astronomy and Informatics, Nicolaus Copernicus University, 87-100 Torun, Poland

**Keywords:** silver nanowire, fluorescence microscopy, plasmonic enhancement, energy propagation

## Abstract

The purpose of this review is to introduce and present the concept of metallic nanowires as building-blocks of plasmonically active structures. In addition to concise description of both the basic physical properties associated with the electron oscillations as well as energy propagation in metallic nanostructures, and methods of fabrication of metallic nanowires, we will demonstrate several key ideas that involve interactions between plasmon excitations and electronic states in surrounding molecules or other emitters. Particular emphasis will be placed on the effects that involve not only plasmonic enhancement or quenching of fluorescence, but also propagation of energy on lengths that exceed the wavelength of light.

## 1. Introduction

Among several critical challenges faced by nanotechnology is development of advanced structures with defined geometry and predictable functions. This involves not only reproducibility of homogenous nanostructure fabrication and synthesis, but also establishing standard, reliable methods of nanostructure manipulation. On the other hand, it is also important to devise new ways of coupling nanostructures and using them for controlled generation and distribution of electromagnetic radiation, which in turn can play a significant role in modulating the optical properties of nearby emitters. Among the types of nanostructures that can be used to influence light concentration and propagation are metallic nanoparticles (NPs), i.e., Particles made of mainly silver, gold, platinum, copper, etc. with sizes in the range of 100 nm. Since such nanoparticles contain free electrons, it is possible to force their collective oscillation which then would yield a local electromagnetic field. Among metallic nanoparticles, particularly intriguing are those with one dimension much larger than 100 nm, as they can facilitate not only localized modification of an electromagnetic field, but can also provide ways to transport energy for distances much longer than the size of diffraction-limited illumination spot. In other words, the scope of this contribution is focused on intermixing plasmon-induced effects, such as enhancement of fluorescence, with plasmon-polariton propagation in metallic nanowires.

The article starts with a brief introduction of the effect of plasmon resonance in metallic nanoparticles followed by a description of basic ideas regarding the interaction between electronic states in optically active nanostructures (dyes, nanocrystals, proteins) and the plasmon excitations in metallic nanoparticles. Distinction between localized surface plasmon resonance characteristic for small nanoparticles and those of surface plasmon polariton present in elongated nanostructures such as metallic nanowires is given. In the main part, three key aspects of using metallic nanowires for assembling hybrid nanostructures are presented, and these include: Fabrication and synthesis of metallic nanowires, examples of influencing the optical properties of various nanomaterials via coupling with plasmon resonance in the nanowires, with particular emphasis on the geometry of a hybrid nanostructure and the spectral properties of constituents, as well as studies of energy propagation in elongated metallic nanostructures. Finally, before a summary and outlook for possible future developments in the field of applying metallic nanowires to different research areas, the example is given of using the nanowires as a geometric and plasmonic platform for sensing the presence of proteins in solution. Demonstrations of both types of benefits related to the geometry of the nanowires and the emergence of the plasmon resonance, underline the advantages such nanostructures bring to the infinite nanoscience and nanotechnology table.

## 2. Plasmon Resonance

### 2.1. Metallic Nanoparticles

When a metallic NP, which is defined as an object with the size less than the wavelength of light, is illuminated with electromagnetic wave, the free electrons within the NP are forced to oscillate. This electron oscillation, called a plasmon resonance, is the source of additional electromagnetic field, which can be used to alter the optical properties of absorbers/emitters placed in the vicinity of such a metallic NP [1]. This unique property of metal NPs is the main reason why these systems have generated great interest in recent years in many, often very diverse research fields, such as optical spectroscopy, photovoltaics, cell imaging, quantum information processing, nanophotonics, and biosensors [2,3,4,5].

**Figure 1:**
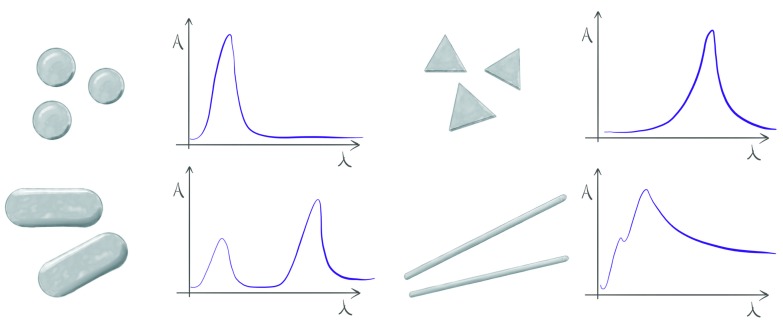
Schematic representation of the influence the morphology of metallic nanoparticles has on the shape of the plasmon resonance.

The optical activity of metallic NPs is determined primarily by the wavelength of the plasmon resonance, which in turn depends on the material, as well as on the NP size, its shape and surrounding environment [6]. This is important, as it allows control of the position of the resonance and for it to be tuned to a particular wavelength range for any given application. Schematic picture of the relation between the morphology of metallic NPs and plasmon resonance is shown in Figure 1. Most common metallic NPs are made of gold and silver and, for spherically shaped NPs, their plasmon wavelengths are around 530 nm and 400 nm, respectively [7], and these values rather weakly depend on the size [7]. A strong shift of the plasmon resonance towards the longer wavelengths can be obtained by fabricating planar triangular nanostructures [8], or nanoshells [9]. Such nanostructures feature indeed a single plasmon resonance, and the spectral range of near-infrared can be easily achieved. On the other hand, nanostructures with elongated shapes, called nanorods, feature two plasmon resonances, one of which corresponds roughly to the resonance observed for spherical nanoparticles of a given material, and the second resonance, whose wavelength is shifted to longer values, and this shift is determined by the aspect ratio of the nanorod [10,11]. With proper control of the fabrication, synthesis and purification, the spectral range of the red-shifted resonance can reach over 1.5 micrometers [11]. This brief description shows that the possibilities of tuning the energy of the plasmon resonance in metallic NPs are virtually infinite and it is feasible to essentially match any wavelength required by any particular application. 

The common denominator of metallic nanoparticles such as spheres, nanorods or nanotriangles is that their sizes are much smaller that the spatial resolution of optical microscopy, which is roughly a few hundreds of nanometers. In contrast, in the case of metallic nanowires, the lengths of which can be as large as tens of microns, it is possible to determine their position on the surface with a simple optical microscope, without the need to apply advanced techniques, such as atomic force microscopy, scanning electron microscopy or transmission electron microscopy. As the nanowires are much longer than the optical resolution of microscopes, it is possible to use these nanostructures as transmitters of energy over distances comparatively larger than the resolution. Still, their diameters are below 200 nm, which makes the nanowires (NWs) plasmonically active, so that both aspects can be combined within a single nanostructure. Furthermore, as schematically shown in Figure 1, the optical spectra of the NWs are very broad, much broader that those of previously described nanostructures. Consequently, we can expect that absorbers/emitters active within the whole visible range will couple efficiently to metallic nanowires. This is particularly valid for nanowires made of silver, characterized with a maximum resonance at around 400 nm. These unique properties of metallic nanowires open qualitatively new ways of implementing them as building-blocks of functional hybrid nanostructures for a variety of applications.

### 2.2. Interaction between Metallic Nanoparticles and Emitters

The optical properties of an emitter placed in the vicinity of a metallic nanoparticle can be affected by plasmon excitations induced by light illumination. Without any metallic nanoparticle, the optical properties of a fluorophore are characterized—in a simplified approach—with three rates: absorption rate, radiative rate, and non-radiative rate. As the plasmon excitation, which originates from collective oscillation of electrons in the metallic nanoparticle, can be considered as an additional local electromagnetic field capable of changing all of the three rates [12,13,14]. In addition, the presence of a metallic nanoparticle can lead to another process related to non-radiative energy transfer from the emitter to the metallic nanoparticle. The influence of plasmon excitations upon the quantum yield of a fluorophore and non-radiative energy transfer between the fluorophore and metallic nanoparticle has recently been studied theoretically [15] with particular focus placed on the dependence on the separation distance between the two nanostructures. For a molecule placed at a varied distance form a metallic nanoparticle with a diameter of 40 nm a strong enhancement of excitation efficiency with decreasing separation was observed. This results from stronger electromagnetic field felt by the fluorophore due to plasmon excitation in the metallic nanoparticle. In contrast, the quantum efficiency exhibits non-monotonic behaviour, and after an initial increase with the reduction of the separation between the emitter and metallic nanoparticle, it starts to decrease significantly, due to the non-radiative energy transfer from the fluorophore to the nanoparticle. In other words, for sufficiently small distances, metallic nanoparticles can absorb any energy and dissipate it in the form of heat. The outcome of these two processes in is a clear non-monotonic dependence of the intensity of fluorescence emitted by the fluorophore upon the distance to the metallic nanoparticle [15]. Importantly, the strongest plasmon-induced enhancement of the fluorescence occurs typically for distances between 10 and 30 nm; for smaller distances non-radiative fluorescence quenching dominates, while for longer distances the fluorophore barely feels the presence of the metallic nanoparticle. Such a behavior has indeed been observed also experimentally, both on ensemble and single emitter levels [15,16,17]. Therefore, by controlling the architecture of the structure, in particular the distance between emitters and metallic nanoparticles as well as their mutual orientation, it is possible to control the interactions in this system and thus to tune the net optical response to any application.

**Figure 2:**
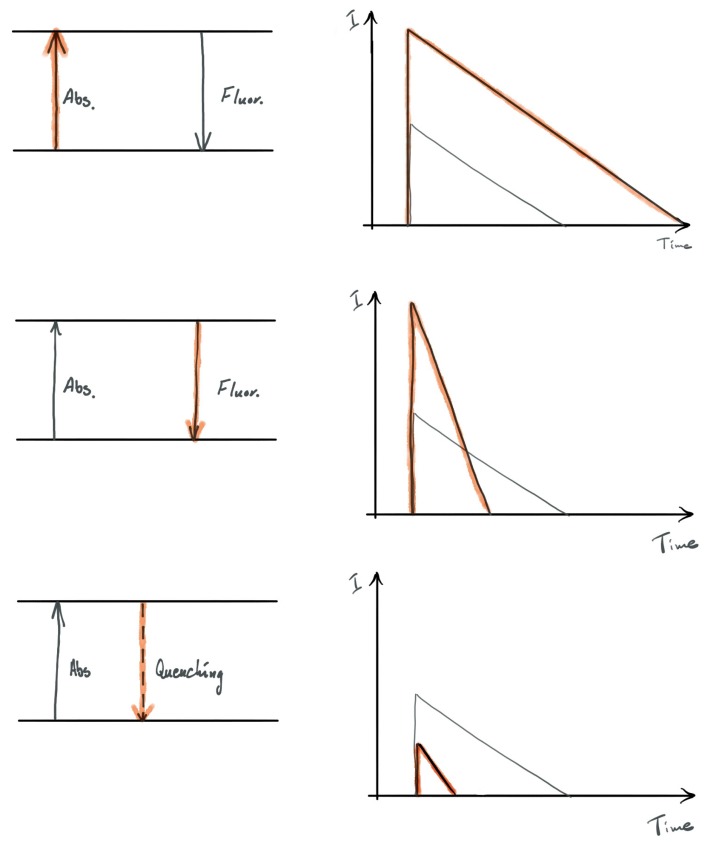
Spectroscopic changes of the optical response of a molecule (nanocrystal, fluorescent protein) coupled to metallic nanoparticle. The rows from top correspond to enhancement of the excitation rate, enhancement of radiative rate, and influence of the non-radiative energy transfer, respectively. Abs and Fluor correspond to absorption and fluorescence, respectively.

Spectroscopically, it is possible to differentiate between the processes that take place between an emitter and metallic nanoparticle, and determine the dominant process. Schematically—for ideal cases—this is displayed in Figure 2, and the scheme relies on the measurement of fluorescence decay characteristic. In the case of absorption rate enhancement, the fluorescence decay remains unchanged, but the overall intensity of emission increases (as shown by the red curve). Next, when the radiative rate is enhanced as the result of plasmon excitations in the metallic nanoparticle, the decay of fluorescence is much faster, and at the same time the emission intensity also increases. Finally, when the dominant effect associated with the presence of a metallic nanoparticle in the vicinity of an emitter is non-radiative energy transfer from the emitter to the nanoparticle, the fluorescence decay is shortened with simultaneous decrease of emission intensity. Although rather simple, this scheme intuitively associates specific interactions to plasmonic nanostructures, and from quite straightforward characterization of the optical properties, allows to conclude about the dominant processes and compare behaviors observed for different systems. 

## 3. Fabrication of Metallic Nanowires

Silver nanowires are 1-dimentional structures with average length in the range of 5 to 100 µm and diameter around 10–200 nm with an aspect ratio greater than 10. They can be obtained by various approaches of either physical or chemical methods. In this section we are going to present a few techniques typically used in the preparation of silver nanowires. Since plasmonic properties are strongly affected by the method by which the silver nanowires were obtained, we will also discuss the advantages and drawbacks of the various synthesis methods. As synthesis of metallic nanostructures is widely studied we present only a summary based on a few selected examples.

### 3.1. Lithography

The most precise method to obtain single as well as an array of metallic nanowire is lithography [18]. This top-down method splits into different types like dip-pen nanolithography, electron beam lithography, focused ion beam, nanoimprinting or photolithography, which all differ in the achievable resolution of the obtain pattern [18]. The methods have in common that they are used to create a pattern in a polymer layer covering the substrate, followed by metal evaporation and finally lift-off, where the polymer is dissolved, leading to nanowire formation within prepared gaps. In the case of photolithography [19], special tricks, such as hydrofluoric acid etching of aluminum masks is needed to reach the desired resolution. Lithography guarantees accurate positioning of the metallic nanostructures on the one hand, but also requires access to sophisticated equipment, expensive chemicals and it is generally quite time consuming. Moreover, the most serious disadvantage of this technique is the polycrystalline morphology of the fabricated metallic nanowires. This dramatically affects the plasmon propagation along the nanowire because of losses via scattering on grains, boundaries and defects.

### 3.2. Wet-Chemistry Synthesis

The most popular method to fabricate metallic nanowires is using wet-chemistry. This is an approach simply based on reduction of metal ions in a solution [20]. At first, the electrochemical techniques were used, in which applied current/potential was used as a reductant, mostly applied in an aqueous solution. Since the obtained samples of nanowires were polydisperse and only a small amount of metallic nanostructure were created, researchers started to growth metallic nanowires in templates. Initially hard templates like nanoporus membranes were based on e.g., anodic aluminum oxide, polymers, silica etc. [7,21]. In the case of hard templates, both electrochemical (reducing agent: applied potential) and chemical reduction (reducing agent: sodium borohydride, alcohols, sodium citrate, ascorbic acid) of metal ions were applied, as well as thermolysis from aqueous solutions, e.g., according to:

AgNO_3_ + NaBH_4_ → Ag + ½ H_2_ + ½ B_2_H_6_ + NaNO_3_ (1)

Although the quality of the fabricated nanowires concerning aspect ratio and sample dispersion is quite satisfactory, the template removal process is harmful for the metallic nanowires causing them to break and degrade, and finally only a small amount of nanowires is obtained. During the development of nanowire synthesis hard templates were replaced by soft ones to overcome the problem of template removal. To this category belongs various polymers (like polyvinyl alcohol, polyethylene glycol, poly(ethylene oxide)-block-poly-(methacrylic acid)) and surfactants based on quaternary ammonium ions and rodlike micelles [22]. The addition of the soft templates leads to anisotropic growth of metallic structure by adsorption at specific crystal surfaces at the same time blocking some and favoring other growth directions. Moreover, some of the soft templates at the same time function as capping agents, preventing the nanostructures from aggregating. It is worth underlining that metal ion reduction in this method is usually performed at room temperature from a seed-mediated solution. Soft-templated methods normally lead to polydispersion of the obtained metallic nanostructures, low aspect ratio and side reactions resulting in a sample rich in nanoparticles.

In further efforts to overcome this issue, the aqueous solution was replaced by ethylene glycol. The advantages of this method were such that is has become seen as distinct from the other wet-chemical methods and is called the polyol method [23]. In this approach to fabricate silver nanowires, a mixture of silver salt with addition of capping and directing growth agent of poly(vinyl pyrrolidone) (PVP) in ethylene glycol (EG) is prepared. Here, EG plays role both as a solvent and reducing agent. EG is oxidized to aldehyde at high temperature which reduces silver ions to metallic silver according to the following reactions [7,24]:

2HOCH_2_CH_2_OH → 2CH_3_CHO + 2H_2_O (2)

or in the presence of oxygen:

2HOCH_2_CH_2_OH + O_2_ → 2HOCH_2_CHO + 2H_2_O (3)

2 Ag^+^ + 2CH_3_CHO → 2Ag + CH_3_COCOCH_3_ + 2H^+^ (4)

**Scheme 1:**
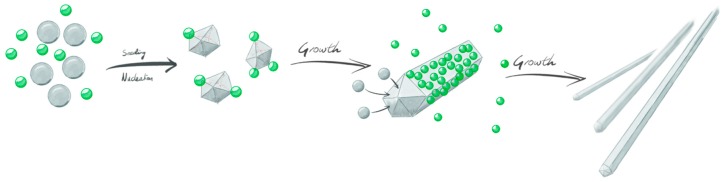
Synthesis of silver nanowires.

While the first silver ions are reduced, the nucleus form fluctuating structures, whose growth can be tuned to obtain required metallic nanostructures. In the case of silver nanowires, during nucleation some multiply twinned particles are formed with exposed {111} and {100} facets. The PVP is specifically and strongly adsorbed on {100} facet causing growth preferentially on the {111} facets leading to elongated structures (see Scheme 1). 

In the polyol method one can vary several parameters to tune the properties of the final metallic structure [7,20]. Among the other reagent concentrations, temperature and reaction time as well as the presence of additives (metallic nanoparticles, halogen ions, oxygen etc.) or the molecular weight of PVP strongly affected the morphology of metallic nanowires and sample dispersion. Using one of the modifications of the polyol method leads to monocrystalline nanowires without defects with controlled dimensions, which is required for plasmon propagation [23].

### 3.3. Printing and Photochemical Methods

Photochemical approach becomes more popular to fabricate single crystal silver nanowires [25]. Simply this method is based on the ultraviolet (UV)-photoirradiation of the sample containing silver salt dispersed in an aqueous solution [25] or in the polymer matrix of e.g., poly(vinyl alcohol) [26]. Recently, for in situ fabrication of metallic nanowires direct writing with a help of laser has become a popular technique [27]. This approach enables to deposit nanowires at different substrates (stiff-glass or flexible-based on polyethylene terephthalate, control morphology and position. To the best of our knowledge no investigation of the last methods was done towards the crystallinity of the nanowires obtained. 

## 4. Metal-Enhanced Fluorescence with Silver Nanowires

The interaction between emitters and metallic nanowires depends primarily on two factors: the optical properties of the emitters, their relation to the nanowire plasmon resonance, and the distance between both components of such hybrid nanostructures. The latter can be rather precisely controlled using bioconjugation, which is based on specific attachment of emitters to metallic nanowires via appropriate linkers. Alternatively, it is also possible to deposit thin dielectric layers that can be considered spacers, on metallic nanowires. This approach, however, is less accurate in particular for the nanowires with diameters larger than 50 nm, as since the spacer thickness should be in the order of a few to a few tens of nanometers to assure efficient coupling, it is highly probable that incomplete coverage of the nanowires will be obtained. Consequently the control of the plasmon interaction with electronic states of the emitters will be limited. The range of reports where the influence of plasmon excitations in metallic nanowires on the optical properties of nearby placed emitters is rather broad [28,29,30,31,32,33,34,35], we shall concentrate here on three types of structures: where emitters are mixed together with the nanowires [29,32] or placed above them [30,31], where conjugation is used to specifically attach emitters to silver nanowires, and where the properties of single emitters are investigated [34,36,37]. This subjective overview will present a variety of structures that can be fabricated and the spectrum of effects that emerge in such systems and that can be studied using fluorescence spectroscopy and spectroscopy.

### 4.1. Planar Structures

The simplest hybrid nanostructure that can be made using emitters and metallic nanowires consists of both components mixed together and deposited on a substrate. The first example of observing coupling between electronic states in emitters and plasmon excitations in silver nanowires involves a water-soluble light-harvesting complex, peridinin-chlorophyll-protein (PCP), which can be found in dinoflagellates *Amphidinium carterae*. The pigment organization of the PCP complex has been determined with 1.3 Å resolution using X-ray crystallography [38], and its optical properties have been studied both on ensemble [39,40] as well as on a single-molecule level [41,42]. The PCP absorption spectrum features an intense, broad band between 400 to 550 nm that is mainly due to peridinin absorption, and two bands at 440 nm (Soret) and 660 nm (Q_Y_) attributed to chlorophyll molecules. The fluorescence emission of the PCP complex originates from weakly coupled chlorophyll molecules and it appears at 670 nm. The optical properties of the PCP complex thus matches rather well with the optical spectrum of the silver nanowires [43]. Together with its simple structure, robustness, and solubility in water, it renders the PCP complex as a stable protein for studying interactions in hybrid nanostructures. 

**Figure 3:**
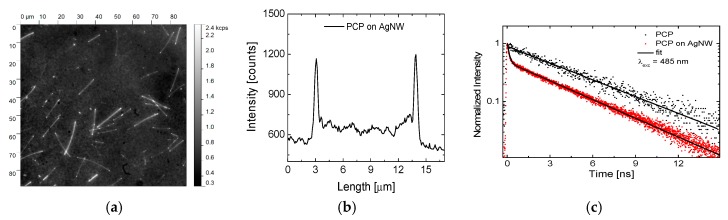
Results of fluorescence imaging and spectroscopy of peridinin-chlorophyll-protein (PCP) complexes mixed with silver nanowires: (**a**) fluorescence map excited at 405 nm, (**b**) intensity cross-section along an exemplary nanowire, and (**c**) comparison of fluorescence decay curves for the excitation spot placed on a nanowire (red) and off the nanowire (black); Lines correspond to fits.

Upon mixing PCP complexes with a solution of silver nanowires synthesized using the polyol method, the sample was deposited on a glass coverslip to enable a fluorescence imaging experiment. The result is shown in Figure 3, where an image measured using 405 nm excitation wavelength is displayed [32]. It features a highly inhomogenous pattern with clear elongated shapes of increased fluorescence intensity. In contrast for a reference sample, where only PCP complexes are present, the image is very uniform and smooth in terms of intensity. Importantly, the fluorescence image seen in Figure 3a can be confronted with a transmission image acquired for the same area of the sample. This yields essentially perfect correspondence between the elongated shapes of increased intensity and the positions of the nanowires on the surface, indicating enhanced emission from the PCP complexes placed in the vicinity of the silver nanowires. Interestingly, the observed plasmonic enhancement overcomes both the contribution of the PCP complexes whose fluorescence emission is quenched (as they are very close to the silver nanowires), as those that are not coupled because of separation that is too far from the nanowires. 

The enhancement of the fluorescence intensity in the vicinity of the silver nanowires can be visualized by extracting a cross-section of the fluorescence intensity along any given nanowire, as shown in Figure 3b. For the displayed nanowire, we observe sharp and spatially narrow peaks at the ends of the nanowire with the emission intensities approximately three times higher than the intensity measured for the PCP complexes off the nanowire as well as along the nanowire. It can be seen, that the intensity on the nanowire is only slightly (typically 20%) enhanced as compared to the area off the nanowire. Analysis of tens of nanowires yields estimation of the enhancement due to coupling with silver nanowires. It turned out that average enhancements obtained for the 405 nm excitation wavelength, which is roughly resonant with the plasmon resonance of the silver nanowires, are equal to 1.32 and 2.4 for PCP located along the nanowires and placed at their ends, respectively [32]. 

However, for a structure, where PCP complexes and silver nanowires are mixed together, the mutual separation ranges from a very small (a few nanometers), where fluorescence quenching is dominant, to a very large (tens of nanometers), where there is no coupling between the emitters and the nanowires. Therefore, fluorescence imaging gives only qualitative information about the enhancement and the measured values cannot be treated quantitatively. While the results of fluorescence imaging provide an excellent way to demonstrate metal-enhanced fluorescence in plasmonic hybrid nanostructures, more quantitative approaches are required in order to understand these effects in greater detail. Such an experiment can be carried out using confocal microscopy, where a laser spot is focused into a diffraction-limited area of the sample. This allows measurement of either fluorescence spectra or fluorescence decays, and the latter in particular can give information about the coupling between emitters and plasmonically active metallic nanostructures. In Figure 3c a fluorescence transient measured for PCP complexes located in the vicinity of the nanowires is compared with that measured far from the nanowires (marked by points). It can be placed on a nanowire as well as off a nanowires. The lines represent bi-exponential and single-exponential fits to the data, respectively. For the PCP complexes deposited on the substrate, the decay constant is of approximately 4 ns [41]. In contrast, for the PCP complexes located along the silver nanowires, two decay times were obtained: the longer comparable to the reference sample and a substantially shorter equal to approximately 0.3 ns. Such a shortening might indicate that the actual enhancement of fluorescence for the PCP complexes coupled to silver nanowires can be over tenfold. The results obtained for hybrid nanostructures composed of the PCP complexes and silver nanowires indicate than elongated metallic nanostructures not only can be used for efficient modification of the optical properties of biologically functional systems, but also more detail can be derived from spectroscopic and fluorescence imaging studies of such structures.

Analogous experiments can be performed for other optically active materials and compounds, including those with potential applications in photovoltaics [44]. In particular, organic electronics is based on polymeric materials and chemical compounds that are considered to be the building blocks of photovoltaic cells and electroluminescent diodes [45]. The optical response of such materials can also be changed by coupling with plasmon excitations in metallic nanoparticles. Moreover, when metallic nanowires are concerned, they can play a bimodal role in such devices: not only by enhancing absorption or emission of organic compounds via plasmon excitations, but also by providing a charge transport network while maintaining relatively high transparency [46,47]. 

Here we consider a structure, where one of the basic materials for organic photovoltaics, a semiconducting polymer poly(3-hexylthiophene) (P3HT), is coupled to silver nanowires [33]. The nanowires were embedded in a conductive polymer poly(3,4-ethylenedioxythiophene):poly (styrene-sulfonate) (PEDOT:PSS) layer, with the P3HT polymer deposited afterwards. All the solutions were spin-coated for 10 sec at 3600 rpm giving a film thickness of undiluted PEDOT:PSS of about 40 nm and P3HT of about 30 nm. Incorporating the nanowires in such a way reduces the efficiency of the non-radiative energy transfer from P3HT, as there is some separation between both materials. Furthermore, this removes any need to transfer the nanowires, synthesized in aqueous solution, to organic solvents, in which P3HT can be diluted, simplifying the overall fabrication process.

**Figure 4:**
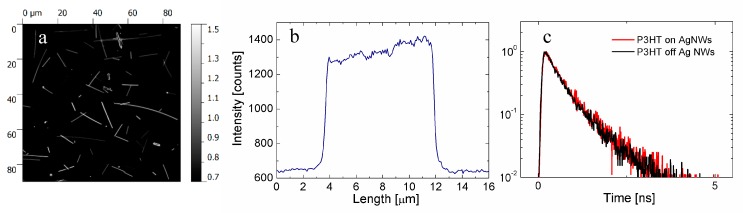
Results of fluorescence imaging and spectroscopy of P3HT mixed with silver nanowires: (**a**) fluorescence map excited at 485 nm, (**b**) intensity cross-section along an exemplary nanowire, and (**c**) comparison of fluorescence decay curves for the excitation spot placed on a nanowire (red) and off the nanowire (black).

In order to characterize the optical properties of P3HT polymer coupled to silver nanowires, analogous set of experiments was carried out as for the structure containing natural photosynthetic complexes, PCP. The fluorescence image obtained using wide-field microscopy is shown in Figure 4a and is filled with elongated shapes characterized with increased fluorescence intensity. Correlating the image with a transmission image measured for the same sample area, where the nanowires can be easily located on the surface, indicates that the stripes of increased intensity occur exactly at positions of silver nanowires. In a similar way to the results discussed before, this observation proves that the optical properties of P3HT can be affected by the presence of silver nanowires, i.e., the interaction with plasmon excitations leads to increase of the fluorescence intensity of P3HT. 

The effect of plasmon excitations upon the fluorescence of P3HT can be quantified by extracting the intensities of P3HT emission on and off the nanowires by making appropriate cross sections. An example of such a cross-section is shown in Figure 4b and it looks rather homogeneous, and also the difference between the intensity measured on the nanowire is much higher compared to the area off the nanowire, in contrast, to what was measured for the PCP complexes. This means that the contribution of molecules that are not coupled to silver nanowires is much less in the case of P3HT, which can be attributed to thinner polymer layers. Indeed, for the thickness in the range of 30 nm (more than an order of magnitude less than in the case of the above-discussed PCP-containing structure, where the thickness was about 200–300 nm), essentially all of the molecules in the vertical direction from the nanowire interact with the plasmon excitation in it. The analysis performed for approximately 100 single nanowires yields the enhancement factor for the P3HT emission equal to 2.1 [33]. 

Time-resolved fluorescence spectroscopy can answer the question about the dominant process responsible for the observed increase of emission intensity [48]. In the case of the hybrid nanostructure containing silver nanowires and P3HT emitter, the observed increase of P3HT emission intensity can be attributed to either an increase of radiative rate or increase of absorption rate, or a combination thereof. In Figure 4c we compare fluorescence transients measured for the P3HT layer deposited on silver nanowires upon excitation at 485 nm. The decay curves measured with a laser spot placed either on (black curve) or off (red curve) the nanowire are identical. Indeed, the emission of P3HT in both cases decays biexponentially with constants of approximately 0.30 ns and 1 ns, as determined using appropriate fitting. The absence of any measurable shortening in the fluorescence decay times of P3HT deposited on the silver nanowires suggests that the enhancement of emission intensity measured using wide-field fluorescence microscopy is a result of an absorption increase of P3HT. The result can be applied in designing organic solar cells with plasmonically improved efficiency.

The availability of various polymers characterized with different absorption and emission spectra, spanning over the whole visible spectral range, from ultraviolet to infrared, allows us to study the influence of plasmon excitations in silver nanowires on the enhancement [49]. Indeed, analogous studies to those described for P3HT were carried out for poly(9,9-di-n-octylfluorenyl-2,7-diyl) (PFO) and poly(9,9-dioctylfluorene-alt-benzothiadiazole) (F8BT) polymers. While F8BT exhibits strong overlap between absorption and emission spectrum with the extinction spectrum of silver nanowires, the other one is active in ultraviolet, where the strength of plasmon resonance characteristic to silver nanowires is much smaller. Consequently, the enhancement of emission in the case of F8BT is comparable to that measured for P3HT, while in the case of PFO it is essentially non-existent. This observation points towards a critical role for prior analysis of the optical spectra of constituents comprising hybrid nanostructures for achieving required functionality.

### 4.2. Bioconjugated Systems

The examples described so far illustrate cases where the control over the morphology of the structure, and thus the influence of plasmonic excitations in silver nanowires on the optical properties of emitters, is rather limited. It is mostly visible in the absence of the ability to define the distance between molecules and silver nanowires. Among the variety of strategies developed for overcoming this limitation, evaporation of optically passive layers with thicknesses comparable to the range of plsmonic interactions (<40 nm), has been frequently employed [16,50]. This enabled the observation of changes of the emission intensity as a function of the distance, as expected from the interplay between the three main processes that emerge in plasmonic systems [15]. However, achieving similar control over the thickness of the emitters, has also been a challenge. All these issues diminish when the structure fabrication is based on the (bio)conjugation approach, where emitters are specifically attached to metallic nanowires, in a similar way as proteins can be deposited on surfaces with the help of proper surface functionalization and/or genetic modifications [51,52,53]. In this way it is possible to achieve defined geometry, where the distance between the proteins and the surface as well as relative orientation are determined by the functionalization method used to bind the proteins. Moreover, just a monolayer of proteins can be attached to the surface, thus by changing the linker used to achieve the conjugation it should also be possible to tune and optimize the influence of plasmonic excitations in metallic nanowires on the optical properties of emitters. 

**Scheme 2:**
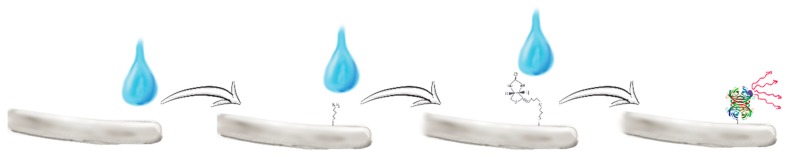
Functionalization of silver nanowires with biotin.

Photosynthetic proteins, such as PCP, are perfectly suitable for conjugation with metallic nanowires, as they can be equipped easily with proper functional groups attached to protein chains. For instance, the PCP complex can be obtained with streptavidin, which in turn exhibits very high binding affinity to biotin [54,55]. Therefore, the step required to obtain bioconjugation in this system is functionalization of metallic nanowires with biotin. The functionalization mechanism used for conjugating emitters with silver nanowires is shown in Scheme 2.

**Figure 5:**
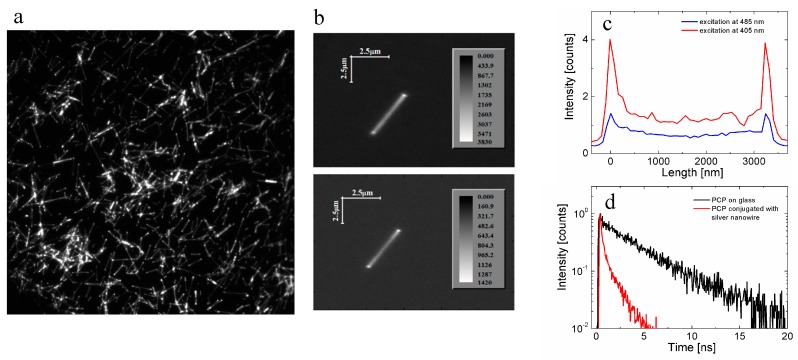
Results of fluorescence imaging and spectroscopy of PCP complexes specifically conjugated with silver nanowires: (**a**) fluorescence map excited at 485 nm of a sample region of 100 µm × 100 µm, (**b**) a pair of maps obtained for the same nanowire for the excitation at 405 nm (upper) and 485 nm (lower), (**c**) intensity cross-sections along the nanowire for both excitation wavelengths, and (**d**) comparison of fluorescence decay curves for the excitation spot placed on a nanowire (red) with the layer of PCP complexes (black).

Formation of a hybrid nanostructure takes place in solution, upon mixing silver nanowires functionalized with biotin together with streptavidin-equipped PCP complexes. Next, the solution containing conjugate can be deposited on a microscope coverslip for evaluating its optical properties. In Figure 5a we show a wide-field fluorescence microscopy image of such a sample, where many elongated bright stripes can be seen, frequently intercrossed, but this is due to a high concentration of nanowires in the solution, and this parameter can be easily controlled during preparation. Nevertheless, by correlating the fluorescence image with the transmission image, where positions of the nanowires can be easily distinguished, it is clear that the emission is associated with PCP complexes attached to silver nanowires. In a clear contrast to the results obtained for a structure, where PCP complexes were mixed unspecifically with silver nanowires, the fluorescence signal comes only from the locations of nanowires, and the areas between the nanowires are dark, which indicates the absence of PCP complexes off the nanowires. This is qualitatively different between the two preparation methods, as bioconjugation substantially improves the control of the morphology of the structure. It is noteworthy that the emission pattern observed for the conjugate also features bright spots at the ends of the nanowires. 

Emergence from the fluorescence emission of the PCP complexes exactly at the locations of the nanowires demonstrates that: (1) PCP complexes attach to the nanowires, and (2) the fluorescence of PCP is not quenched. However, it cannot be used to argue that both nanostructures interact with each other, let alone that the optical properties of the PCP complexes are affected by the plasmon excitation in silver nanowires. The absence of the reference requires devising another way of proving that there is coupling between electronic states in emitters and plasmon excitations in silver nanowires. The idea relies on the fact, that the emergence of spectrally resonant interaction in a system should influence the overall spectral properties of this system. In other words, the optical response of an isolated emitter should be qualitatively different as compared to this emitter coupled plasmonically to metallic nanowire. This property is visualized in Figure 5b, where the fluorescence images of a single silver nanowire with PCP complexes attached is measured with two different excitation wavelengths: 405 nm and 480 nm. The first one corresponds to the maximum of plasmon resonance in silver nanowires, while the other is the maximum of absorption of PCP complexes. For isolated PCP complexes, or complexes that are not experiencing plasmonic influence from the nanowires, the fluorescence intensity measured for 485 nm excitation should be substantially higher than that measured for 405 nm excitation. Interestingly, the result displayed in Figure 5b is qualitatively different: excitation at 405 nm yields much stronger emission of the PCP complexes. This is further demonstrated by cross-sections obtained for this nanowire, indeed the emission for the wavelength of 405 nm is twofold higher than that of 485 nm Figure 5c. Consequently, even in the absence of a direct reference, the ability to probe wavelength dependence of the optical properties of a hybrid nanostructure allows to elucidate both the emergence and the scale of plasmonic interactions. 

Of course in order to quantify the effect of plasmon excitations it is also possible to measure fluorescence dynamics. In the case of the PCP conjugate on silver nanowires, the result of such an experiment carried out on a single nanowire is shown in Figure 5d. Comparison between the fluorescence decay curve measured for the conjugated sample and the reference points towards strong shortening of the former, which unambiguously confirms efficient coupling between the protein and the silver nanowire. Notably, this decay curve is also qualitatively different from that obtained for a planar sample, where PCP complexes were just mixed with the nanowires, as it features no long decay clearly visible for the mixed sample. This result reinforces the conclusion that upon bioconjugation it is possible to obtain hybrid nanostructures with much better uniformity, both geometry-wise and interaction-wise. 

Moreover, the combination of fluorescence imaging and time-resolved fluorescence microscopy undoubtedly confirms the presence of plasmonic interactions in the structure. There are however cases, in particular those, where the primary mechanism of fluorescence enhancement is associated with the increase of the absorption rate, and where the changes of fluorescence decay are minimal or difficult to interpret [36]. In such instances the only remaining approach to prove the emergence of plasmonic coupling between emitters and nanowires should be based on wavelength-dependent imaging. 

In the context of bioconjugated hybrid nanostructures, it is important to consider the universality of this approach, and discuss to what extent it is possible to extrapolate the results obtained for a given emitter in terms of expecting similar results. In order to address this issue it is necessary to repeat experiments with conjugated samples, but using a different emitter. Semiconductor quantum dots [56] are considered to be highly promising structures for a variety of applications, thus enhancing their optical response via plasmonic interactions would be of high interest [54,55,57]. Furthermore, these nanostructures can be synthesized from many materials with a variety of ligands/functional groups at the surface, facilitating assembling conjugated structures in a relatively straightforward way. On the other hand, the conjugation of nanostructures is a process that can be monitored in real time [54], giving insight into the dynamics of this effect. The experimental approach applied to study real-time conjugation of semiconductor quantum dots made of CdTe with silver nanowires in solution required an experimental setup where, simultaneously, emission spectra and decay curves can be measured. The experimental configuration is similar to that described in [54], however in addition to collecting the emission spectra it also allows to obtain information about fluorescence dynamics, which is vital for assessing the nature of interaction between emitters (quantum dots) and metallic nanostructures. Importantly, the silver nanowires used in this experiment were similar to the ones used for conjugating the PCP complexes, and the conjugation protocol was identical, and based on streptavidin-biotin linkage. 

**Figure 6:**
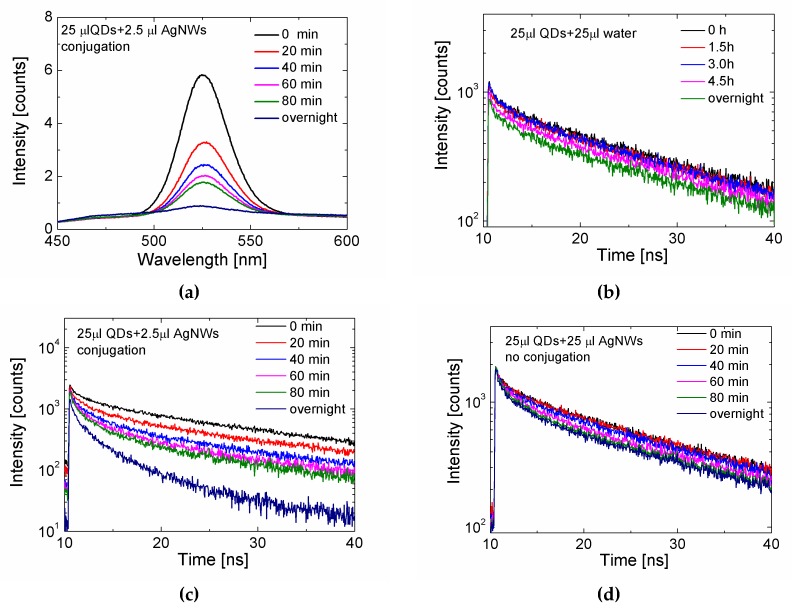
Results of fluorescence spectroscopy of conjugation between CdTe nanocrystals and silver nanowires: evolution of fluorescence spectra (**a**) and fluorescence decay curves for a mixture between the nanocrystals and functionalized nanowires (**c**). Fluorescence transients collected for both solution of nanocrystals-only (**b**) and a mixture of nanocrystals and nanowires without functional groups are shown in the (**d**).

The result of such an experiment is displayed in Figure 6, where in addition to the dynamics of fluorescence spectrum and decay measured for a conjugate between CdTe nanocrystals and silver nanowires (Figure 6a,b), also reference measurements are shown. The latter include temporal evolution measured for a solution of CdTe quantum dots only (Figure 6c) and for a solution of CdTe quantum dots mixed with silver nanowires without the biotin attached, i.e., where no conjugation is expected (Figure 6d). For both reference samples, the emission spectra and fluorescence decay curves are constant over time, in fact for the solution of CdTe quantum dots the change is minimal even after overnight, which indicates high photostability of these nanostructures. Adding silver nanowires with no biotin attached to the solution of quantum dots results in essentially no change of emission intensity and decay within 80 min from the start of the experiment. It might suggest that the electronic states of quantum dots exhibit no influence of plasmon excitations in silver nanowires. Since the nanowires are not functionalized with biotin, which would enable specific binding between them and the nanocrystals, the contact and the chance for interaction is sporadic and based in principle on collisions. 

In contrast for a solution mixture where CdTe quantum dots can bind specifically and permanently to silver nanowires, the spectral changes both in the spectra and emission decays are observed. In fact, upon adding silver nanowires to the solution of CdTe quantum dots the emission intensity decreases continuously with time, and after 80 min it is barely detectable. This reduction in emission intensity is accompanied with the shortening of fluorescence decay time. Interestingly, this experimental approach allows us to obtain insights into the dynamics of conjugation, as it can be assumed that when there is no fluorescence of the sample, all of the quantum dots are attached to silver nanowires. Indeed, for a mixture where the concentration of quantum dots used was lower, the decrease of emission intensity with time progressed much more rapidly. Taking both effects into account, it can be concluded that in this hybrid nanostructure the dominant effect is associated with the non-radiative energy transfer from the quantum dots to silver nanowires, which then results in strong and efficient quenching of the emission. 

Comparison of the two experiments, where the same conjugation scheme between emitters and silver nanowires yielded qualitatively different outcome, is important in understanding that it is not possible to directly extrapolate the results obtained for one plasmonic hybrid nanostructure to another one, although the difference might seem minimal at first glance (in this case it concerns changing the emitter). Qualitative explanation of the observed behavior is based on the difference between a protein-shielded organic molecule, which in the case of the PCP complex is chlorophyll, and the quantum dot, where the exciton Bohr radius [56] is generally larger than the size of the dot itself. Therefore, while in the case of the PCP complex the excitation on chlorophyll molecule is tightly localized and, furthermore, shielded from the surroundings, the exciton in a quantum dot is not only exposed to it. In addition to the leakage of exciton wavefunction, one can expect the effective distance to be much shorter in the case of CdTe quantum dot conjugated to silver nanowires, which might explain the dominant contribution of the non-radiative energy transfer leading to complete quenching of the dot emission with time. 

In summary, interaction between emitters and metallic nanowires is a complex process and the effects observed on such structures depend not only on the type of emitters used for constructing a hybrid plasmonic nanostructure, or on the way to attach emitters to silver nanowires. By contrast, each case has to be analyzed separately as the results vary both in regard to the net outcome (emission enhancement or quenching) and the actual mechanism associated with it (increase of absorption rate or radiative rate). Nonetheless, application of silver nanowires for enhancing the optical response of emitters is highly promising for detecting proteins or improving the functionality of photovoltaic and optoelectronic devices.

## 5. Propagation of Energy in Metallic Nanowires

Metal-enhanced fluorescence is characteristic of metallic nanoparticles, such as spherical particles, nanorods or nanotriangles, but in order to propagate energy over large distances it is necessary to use metallic nanowires, whose sizes in one dimension extend over a micron. In combination with standard spatial resolution of optical fluorescent microscope (300–500 nm) such lengths are sufficient to observe propagation of energy upon light illumination [58,59,60,61,62]. At the same time, metallic nanowires can facilitate plasmon enhancement of the optical response of emitters placed nearby. Bringing together these two functionalities open highly attractive possibilities to control light at the nanoscale, also in the context of remote addressing and probing the optical properties. In most experiments aimed at understanding the propagation of energy in metallic nanowires the configuration was based on localized excitation of one of the ends of a nanowire and wide-field monitoring of the resulting emission [60,62]. Moreover, the emitters used for that purpose were excited directly, using one-photon excitation. 

The results of energy propagation in metallic nanowires described in this review were obtained in a different system since, as luminescence probes, up-converting nanocrystals were applied [63,64]. Such materials exhibit efficient luminescence in visible spectral range upon excitation in near infrared spectral range. This emission is associated with erbium/ytterbium ions incorporated into fluoride matrixes [65]. The sizes of these nanocrystals can be of the order of 20 nm. 

**Figure 7:**
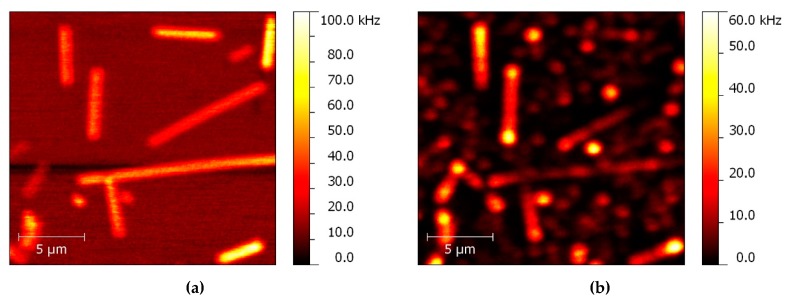
(**a**) Scattering image obtained for silver nanowires coated with up-converting nanocrystals, and (**b**) luminescence map collected for the same sample area upon excitation at 980 nm and detection at 650 nm.

The first indication of energy propagation in a system combining silver nanowires and up-converting nanocrystals can be concluded from the results of a standard luminescence imaging experiment, shown in Figure 7. Before acquiring luminescence image, a scattering pattern was measured (Figure 7a) where the positions of silver nanowires on the substrate surface can be precisely determined. When on such a substrate up-converting nanocrystals are deposited, and at the concentration that allows probing the optical properties of individual nanostructures, the luminescence image shown in Figure 7b can be obtained. The excitation wavelength of 980 nm and the detection wavelength of 650 nm were used. There are two types of structures that can be distinguished in the image. First of all, in the background a number of spots with moderate intensity appears and the positions of these spots show no correlation with the positions of silver nanowires as seen in the scattering image. This emission is attributed to luminescence of single up-converting nanocrystals that are far away from the nanowires. On the other hand, there are elongated shapes visible in the image, whose positions are perfectly correlated with the locations of silver nanowires. The emission intensity associated with the nanowires is considerably stronger than that of single, isolated nanocrystals, most probably due to coupling between electronic states in the nanocrystals and plasmon excitations in silver nanowires [64]. On average the enhancement factor in such structures approaches 6, but it can vary from nanocrystal to nanocrystal. 

However, a more surprising observation is related to the fact that in most cases whole nanowires are emitting light. The first and the most straightforward explanation would rely on the fact that there are nanocrystals everywhere on the substrate and, for a confocal microscopy image, in every spot the nanocrystals are excited with the laser. However, this is not the case with this sample. Indeed, the concentration of nanocrystals on the surface is very low, in order to enable imaging luminescence of individual nanocrystals. Consequently, the opposite is highly probable: that no nanocrystals are excited within the laser spot at many locations across the sample. When in addition the laser spot is placed in the area where no nanowires are present, no emission is observed. By contrast, when a nanowire is illuminated then from the image shown in Figure 7b it can be inferred that regardless whether there are also nanocrystals within the laser spot or not, the optical response can be detected. In this scenario also two possibilities must be considered: simultaneous excitation of a nanowire and placed nearby nanocrystals, and excitation of a nanowire only. In the first case the emission is observed, which corresponds to the emission excited in the nanocrystal coupled to the nanowire. In the second case detection of light indicates that the infrared excitation at 980 nm excited propagating plasmon-polaritons in a silver nanowire, which in turn is capable to excite up-converted emission of the nanocrystals that are in the proximity of the nanowire, but not excited directly with the laser. Furthermore, the emission of these nanocrystals couples back to the nanowire, exciting plasmon polaritons but with energies corresponding to the emission wavelengths characteristic for the nanocrystals, and this emission is observed at the location of the nanowire excited with the laser. In summary, the observation of luminescence across the whole nanowire implies that metallic nanowires can be used for propagating energy over distances much longer than the size of the laser spot, which is of the order of half a micron. 

**Figure 8:**
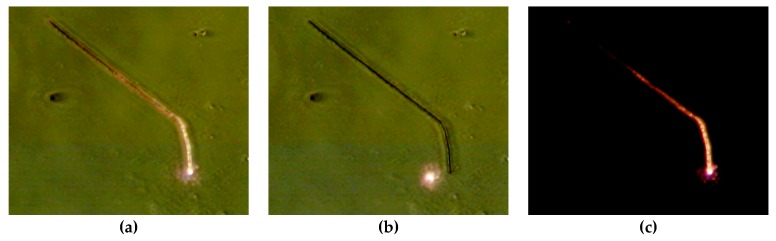
Images of the structure composed of a silver nanowire covered with a layer of up-converting nanocrystals. By controlling the position of the laser spot (980 nm) it is possible to demonstrate energy propagation in a metallic nanowire: (**a**) laser placed on the nanowire, (**b**) laser placed off the nanowire, (**c**) luminescence image measured in configuration (**a**).

Experimental demonstration of this effect can be made using a microscopy setup where the excitation is provided by a laser and is focused on a diffraction limited spot, while observation is carried out in a wide-field geometry, where large area of the sample is imaged at once. An example of the result of such an experiment, where up-converting nanocrystals were deposited on a curved silver nanowire, is shown in Figure 8. The position of the excitation laser spot can be smoothly varied across the sample surface, thus it is possible to place it either on the nanowire or off it, and the position can be directly seen on a camera (Figure 8a). Importantly, the detection is not spectrally selective, so that both main bands contribute to the emission image. When the laser excites only nanocrystals, one can distinguish spotty emission patterns that can be attributed to the luminescence of the nanocrystals (Figure 8b). In particular, when white-light illumination is switched off, the luminescence pattern associated with nanocrystals can clearly be seen. On the other hand, placing the laser spot exactly at one of the nanowire ends leads to intense brightening of the nanowire, which is approximately 20 µm long (Figure 8c). Indeed, the energy excited by infrared laser excitation propagates efficiently in the nanowire, despite the fact, that this particular nanowire is not straight but rather curved. It is important to reiterate the sequence of events that must take place in order for such a result to be observed. First the infrared laser excites nanocrystals, where the up-conversion process transforms infrared photons to visible ones. In the second step the emission of the nanocrystals should couple to the nanowire and excite surface plasmon polariton, which propagates along the nanowire. 

This result implies that plasmonically active elongated metallic nanowires can be used for rather efficient transport of energy over distances reaching tens of microns. In particular, since this energy can be further used for the activation of emissions, it is straightforward to imagine that also photoactivation of proteins [66], such as green fluorescent proteins or photocatalysts should be possible in such a scheme [67]. In a way this concept can combine benefits associated with plasmonic enhancement with remote efficient photoactivation, for more efficient catalysis, sensing or optoelectronics. 

## 6. Biochemical Sensing with Metallic Nanowires

**Figure 9:**
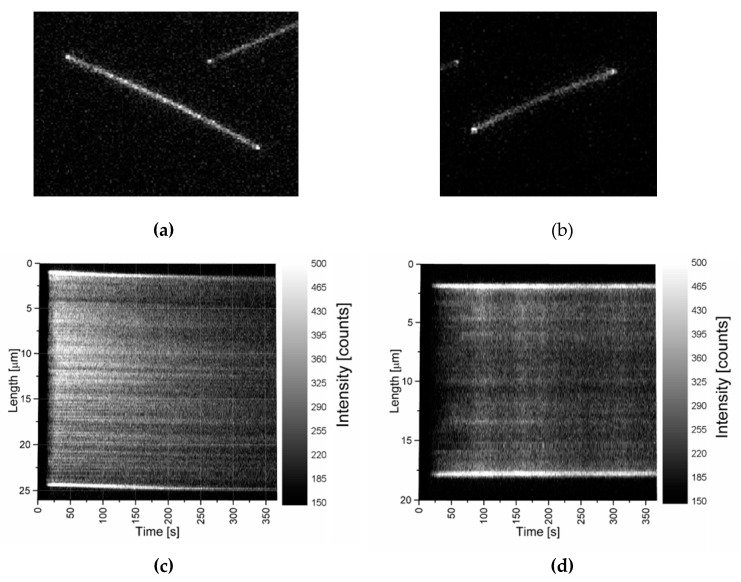
Temporal evolution of fluorescence for a single nanowire covered with a solution of PCP complexes. The nanowire on a glass substrate is shown in the left column, while the right column shown the results obtained for the nanowires deposited on a streptavidin-coated glass substrate. Images of fluorescence for proteins attached to silver nanowires are shown in (**a**) and (**b**), respectively, while the evolutions of fluorescence are displayed in (**c**) and (**d**), respectively.

The metallic nanowires described above due to their optical properties can be used for (bio)chemical sensing. Because they are visible under optical microscope they are perfect candidates to be a geometric platform to study molecular interaction. This approach was used to study the immunocomplex formation between antibodies immobilized on the silver nanowires and viruses by correlating transmission and fluorescence images obtained after depositing nanowire suspension on a substrate. Additionally, a dynamic search for viruses by mixing antibody-modified nanowires in solution substantially increased the probability for immunocomplex formation, and created the possibility to reach a medically relevant detection level of 10^3^ pfu/mL [68]. Since the nanowires are also plasmonic nanostructures one can used the electromagnetic field to influence fluorescent properties of molecules present in the close vicinity of metallic nanostructures [37]. Herein, we would like to show all of these remarkable optical properties to present real-time determination of protein with a help of AgNWs. Additionally, we would like to underline how important surface modification is and can affect the properties of the fluorescence protein. For real-time measurements of PCP on silver nanowires, wide-field fluorescence microscopy was used. The spin-coated biotin-modified AgNWs were localized on the cover slip first in transmission mode, and secondly inspected in the fluorescence one to measure the reference signal. Next, the drop containing PCP was cast on the sample and fluorescence were constantly measured (Figure 9). The time-traces of fluorescence intensity shows that 0.5 s is enough for streptavidin-tagged PCP to bind with biotin anchored on AgNWs and signal saturation is observed after 12.5 s, followed by the decrease of the signal intensity caused by the photobleaching process. The dynamic of the conjugation process between PCP and biotin is strongly influenced by the cover slip modification. Namely, when the glass was first modified with streptavidin and next the biotin-modified AgNWs were deposited the conjugation spread in time and the signal saturation is reached within 20 s. Although, the intensity of the fluorescence is smaller in the case of modified glass, the photobleaching is much smaller and no fluorescence of PCP out of AgNWs is observed. The fluorescence enhancement for both modified and unmodified glass is similar. This show significant impact of surface modification on the molecular recognition. In principle such a system can be really useful to study molecular interaction down to a single molecule. 

## 7. Summary and Outlook

Metallic nanowires combine three properties that make these nanostructures truly unique in the context of controlling the optical properties of molecules, nanocrystals, and proteins. Their lengths in the order to tens of microns enable direct imaging with optical microscopes, both in transmission and fluorescence modes. Thus, by correlating the two images it is possible to conclude with interactions between emitters and nanowires. Second, the nanowires are plasmonically active structures, which can be used for influencing the electronic states in emitters placed nearby. Depending on the actual architecture, either fluorescence enhancement or fluorescence quenching can be observed, and both scenarios can have an impact on designing new sensors and more efficient photovoltaic platforms. Last but not least, by proper illumination it is possible to inject energy into the nanowires and this energy can be transported over large distances, possibly activating molecules placed at remote locations away from the excitation spot. 

This review article showed several examples that are expected to form a scaffold for devising future directions of metallic nanowire research. We believe it can serve as both the background description of basic effects, as well as insightful inspiration for research projects. Among the key challenges faced at present are the ability to synthesize long nanowires, with lengths in the order of a millimeter, which could bridge the nanoworld with the macroworld, in order to position nanowires precisely on surfaces for fabricating matrices with defined geometry, and to develop more efficient ways to control the surface properties of the nanowires for tailoring their biofunctionality.
